# Supplementary material for: People Can Create Iconic Vocalizations to Communicate Various Meanings to Naïve Listeners
Source: Sci Rep. 2018 Feb 8;8:2634. doi: 10.1038/s41598-018-20961-6 (PMC5805706; doi:10.1038/s41598-018-20961-6)
Supplement: Supplementary file 1 — Vocalization rationales [file 41598_2018_20961_MOESM1_ESM.docx]

**Supplementary Information for:**

**People Can Create Iconic Vocalizations to Communicate Various Meanings to Naïve Listeners**

Marcus Perlman^1^

Gary Lupyan^2^

1. Department of English Language and Applied Linguistics, University of Birmingham
2. Department of Psychology, University of Wisconsin-Madison

Corresponding author:

Marcus Perlman

m.perlman@bham.ac.uk

Supplementary Table 1. Vocalization rationales included with the contest submissions.

| Team | Meaning | Rationale |
| --- | --- | --- |
| AB | bad | Falling tone |
| AB | big | Intuited |
| AB | child | More affectionate sounding |
| AB | cook | Intuited |
| AB | cut | allows air to pass which mimics feeling of cutting through something |
| AB | deer | Passive |
| AB | dull | round sounding |
| AB | eat | Intuited |
| AB | fire | Similar to ‘cook’ |
| AB | fruit | Intuited |
| AB | gather | Intuited |
| AB | good | Rising tone |
| AB | hide | passive sounding |
| AB | hunt | Intuited |
| AB | knife | Similar to ‘cut’ |
| AB | man | Intuited |
| AB | many | Intuited |
| AB | meat | Intuited |
| AB | one | Intuited |
| AB | pound | Percussive |
| AB | rock | Intuited |
| AB | sharp | similar to ‘cut’ |
| AB | sleep | non-percussive |
| AB | small | Intuited |
| AB | snake | Aggressive, Indirect |
| AB | that | similar to ‘this’ |
| AB | this | Intuited |
| AB | tiger | Aggressive, Direct |
| AB | water | Intuited |
| AB | woman | Softer |
| BW | bad | the sound I would make when I am disgusted; the diphthong makes it sound ugly and abnormal |
| BW | big | long, low vowel with low pitch to indicate large size |
| BW | child | my attempt at imitating a child, high-pitched (since children are small) |
| BW | cook | cooking is complicated, so this vocalization has many parts, including the use of my tongue to make a 'wet' sound |
| BW | cut | high-pitched sound that is repeated, since cutting is a repeated action |
| BW | deer | my attempt at imitating a deer, low pitched, animalistic sounding |
| BW | dull | the most boring sound I could make, long and even (minimal modulation of my voice), like a dull surface |
| BW | eat | the sound somebody makes when they eat with pleasure and quickly, making a 'wet' sound with the tongue and slurping; also a complex sound because eating is a complex elongated action |
| BW | fire | sound with high timbre like the sound of fire wood cracking |
| BW | fruit | similar to eat but more high-pitched because fruit are sweet |
| BW | gather | gathering involves many things, so I made this sound repeated; and gathering to me is something we like to do quickly, so I made those sounds quick and a bit high-pitched, since when we gather things, we are generally happy? |
| BW | good | fully vocalic, sonorous and loud indicates good quality and satisfaction |
| BW | hide | a gasp of air as being afraid, which makes you want to hide |
| BW | hunt | like a war cry, has a rhythmic up-beat structure to it that is good for shouting in groups |
| BW | knife | the sound of a knife hitting the air, voiceless because knifes are small and light, repeated because I imagined hitting somebody multiple times |
| BW | man | low-pitched and self-satisfied sound |
| BW | many | many is the same sound repeated |
| BW | meat | to stone-age people, eating meat must have been very satisfying, so this vocalization sounds happy and evolves smacking sounds reminiscent of my eat vocalization |
| BW | one | the same sound as 'many' but just once; also short in duration because 'one' is a small magnitude |
| BW | pound | pounding is loud and noisy, so this is a noisy sound; it's also a repeated action, hence this is repeated |
| BW | rock | although this may sound like a bee, it is actually a rock! rocks are boring and don't move so this is an extended vocalization, but rocks don't have a smooth surface but feel rough, so there's a little modulation in there as well |
| BW | sharp | sharp things hurt, so I made the hissing sound humans commonly do when they get hurt |
| BW | sleep | a snoring sound involving the full beauty of my nostrils |
| BW | small | small objects are generally high-pitched and so is this sound |
| BW | snake | some snakes hiss hence the hissing; also very similar to my sharp vocalization, which makes sense because snakes can hurt and have sharp teeth |
| BW | that | I have absolutely no justification for this one, except that this was what came to mind most readily when I simultaneously pointed to a close distance or a far distance |
| BW | this | I have absolutely no justification for this one, except that this was what came to mind most readily when I simultaneously pointed to a close distance or a far distance |
| BW | tiger | my attempt at imitating a tiger, with a bit of a roar and some voiceless elements I guess because tigers are light and fast? |
| BW | water | the smacking sounds 'wet' and the preceding voiceless sounds have a wavy quality to them, like water |
| BW | woman | women have high-pitched voices, so this is the analogue of the male vocalization, although perhaps less self-satisfied and a bit surprised sounding |
| GB | bad | A raw voice saying a short sharp non-word in a scolding tone |
| GB | big | Long open vowels and 'chubby' round consonants, ending in vowel to make it more adjective-like |
| GB | child | Kind of whiny high pitch voice non-word and a little sneeze |
| GB | cook | Sounding like making fire and then boiling water |
| GB | cut | Sharp sounds, reduplicated for verb-likeness |
| GB | deer | A non-existing morpheme meaning 'animal' and clicking hoofs |
| GB | dull | Softish consonants and vowels that make it sound boring and monotone, ending in vowel to make it more adjective-like |
| GB | eat | Sounds of chewing and swallowing, reduplicated for verb-likeness |
| GB | fire | A non-existing morpheme meaning 'in-animate thing' and sounds of starting a fire, blowing air and sizzling |
| GB | fruit | A non-existing morpheme meaning 'in-animate thing' and a bite plus slurp as if eating something juicy |
| GB | gather | A voice calling in a manner that sounds like gathering people together and some sounds that imitate something being plucked from a tree and falling in a basket, all reduplicated for verb-likeness |
| GB | good | A singing voice as if a bright light shines down from the sky and all is going very well |
| GB | hide | A sound of someone frightened and in danger and trying to get away while trying to not be too loud, all reduplicated for verb-likeness |
| GB | hunt | A howling dog, arrows being fired (reduplicated) and an animal whining |
| GB | knife | A non-existing morpheme meaning 'in-animate thing' and the sharp sound used for 'cut' |
| GB | man | Non-sense speech-like sounds in a male voice |
| GB | many | Sounds as something bouncy, repeated short syllables |
| GB | meat | Some domesticated animal sounds and chewing |
| GB | one | A single short nonsense syllable |
| GB | pound | Short, bursty consonants and vowels with a 'resonating' quality, reduplicated for verb-likeness |
| GB | rock | A non-existing morpheme meaning 'in-animate thing' and the sound for pound |
| GB | sharp | High/front vowels, consonants that imitate cutting sounds, ending in vowel to make it more adjective-like |
| GB | sleep | Snoring and whistle-y breathing |
| GB | small | A tiny sneeze |
| GB | snake | A non-existing morpheme meaning 'animal' and a sort of uvular fricative + high/front vowel combination |
| GB | that | Nonsense syllables with low/back vowels imitating far-away-ness |
| GB | this | Nonsense syllables with high/front vowels imitating closeness |
| GB | tiger | A non-existing morpheme meaning 'animal', a snarl and a purrrrr |
| GB | water | A non-existing morpheme meaning 'in-animate thing' and sound of water drops |
| GB | woman | Non-sense speech-like sounds in a female voice |
| IL | bad | sounds of disgust/ dislike |
| IL | big | long, drawn out, rounded, forceful sound to evoke associations of largeness and weight |
| IL | child | crying/ whining baby sound |
| IL | cook | sound of a fire crackling combined with an iconic sound of eating |
| IL | cut | imitating the sound of a scissors or a saw-like sound; repeated to emphasize that it is an action (not, e.g., the knife) |
| IL | deer | sound of hooves clopping combined with a benign, soft sound; since deer is the only hoofed, non-predatory animal, this sound is fairly unspecific and meant to contrast with the other animal sounds |
| IL | dull | low, monotonic, lax vowel sound |
| IL | eat | sound of slurping/ chewing/ lips smacking; the slurping sound is added to try to create a contrast to eating-related things such as ""fruit"" and ""meat"" |
| IL | fire | imitating the sound of fire starting and crackling |
| IL | fruit | imitating the sound of biting a piece of apple along with a pleased sound to signify sweetness |
| IL | gather | what we went for here is to indicate some sort of decision/ choice or discovery with the pensive or perplexed ""mmmm"" sound followed by a triumphant sound; the final collection of little sounds is supposed to poiunt to the process of collecting (with repeating sounds again pointing towards actions) |
| IL | good | a sung major fourth to indicate something that is emotionally positive/ triumphant |
| IL | hide | a suppressed gasp of surprise or fear followed by a kind of hushed trembling sound of fear; supposed to evoke the image of someone hiding while trembling in fear |
| IL | hunt | imitating gunshots |
| IL | knife | imitating the sound of a blade whooshing through the air followed by a cutting or sharp sound; we chose whooshing specifically in the hope of contrasting our ""knife"" sound with our ""cut"" sound |
| IL | man | very manly (low, gruff) grunts (two so it can't be confused with ""one"") |
| IL | many | an accumulation of short, simple, neutral vocalizations |
| IL | meat | lip-smacking/ chewing sounds that are supposed to simulate a more effortful, tearing process that would be necessary for eating meat; this is to try to distinguish this sound from ""eating"" and ""fruit"" |
| IL | one | single simple, neutral, short vocalization (as long as this sound is neutral and short, it can't really be confused with any of the other meanings) |
| IL | pound | repeated dull, impact-evoking sound |
| IL | rock | a dull sound, perhaps the sound of a rock hitting the ground; only one short sound, since repeating these sounds is reserved for actions (e.g. ""pound"") |
| IL | sharp | short, high-pitched, ""eeee"" sound (associated with sharpness, pointiness) |
| IL | sleep | imitating the sound of snoring |
| IL | small | small, short, high , simple sound, mean to contrast with the long, drawn out, loud sound for big |
| IL | snake | imitating the sound of a snake flitting its tongue in and out of its mouth |
| IL | that | imitating a call to something/ someone far way; ""that"" as being spatially far |
| IL | this | a gibberish sound evoking something like ""right here"". The inflection is meant to be contrastive, as if the speaker were trying to distinguish one thing from another (""not that one, THIS one""); this inflection also helps distinguish ""this"" from ""good"", e.g. |
| IL | tiger | imitating the sound of tiger growling/ roaring |
| IL | water | sound of gulping or a mock sound of a fish 'gurgling' underwater |
| IL | woman | stereotypical sound of a young woman fake-giggling/ laughing |
| KD | bad | complementary to the above [good] |
| KD | big | complementary to the above [small] |
| KD | child | an otherwise underspecified fast, variably high-pitched human vocalisation |
| KD | cook | {fire} {eat} |
| KD | cut | {knife} {knife} {knife} - repeated use of a knife |
| KD | deer | snickering followed by hoof clattering |
| KD | dull | sound produced by soft, lax lips to indicate non-sharpness |
| KD | eat | mixed bilabial + alveolar clicks - stylised chewing noises |
| KD | fire | sound of wood burning at very high temperature (also similar to a furnace or gas oven) |
| KD | fruit | [kk] {eat} - crunching sound followed by eating |
| KD | gather | {many} {eat} |
| KD | good | indicating excitement, also cf. the 'up is good' metaphor |
| KD | hide | ingressive - indication of surprise which is partly suppressed to avoid drawing attention of the cause of the surprise |
| KD | hunt | loud/aggressive battle scream |
| KD | knife | sharp cutting noise |
| KD | man | an otherwise underspecified low-pitched human vocalisation |
| KD | many | {one} {one} {one} - multiple non-specific counting clicks |
| KD | meat | {deer} {eat} |
| KD | one | minimal, as non-referential/non-specific as possible 'counting' click |
| KD | pound | {rock} {rock} {rock} - repeated use of a rock |
| KD | rock | sound of a dull, heavy object hitting an equally dull/soft surface |
| KD | sharp | any fricative with a high spectral centroid would be iconic of sharpness/cutting sounds - since [C]/[x] are already used for knife/cut as well as fire, and the probably most iconic [s] is ruled out on snakey vocal emblem grounds, the remaining best choice might be [S] (without the final [t] to indicate only the essential property of sharpness) |
| KD | sleep | mixed in+exgressive - stylised rendering of snoring |
| KD | small | high pitch, as would be produced by short (small) oscillating objects, combined with a vowel with most spectral energy in higher frequency bands, indicative of a small resonating chamber |
| KD | snake | tongue-flapping while breathing in - akin to a snake's hissing |
| KD | that | {one} - long stretched vowel to indicate distance to the non-specific referent indicated by {one} |
| KD | this | {one} - complementary to the above, with a shorter vowel |
| KD | tiger | roar of a tiger |
| KD | water | a dripping sound |
| KD | woman | an otherwise underspecified high-pitched human vocalisation |
| KR | bad | spoken with negative affect, phonemes that make face into unhappy shape, slow, low F0. |
| KR | big | low F0 |
| KR | child | highest F0 with similar pseudosyllable structure to the other people words. |
| KR | cook | Trying to capture the noisy high frequencies of the sizzle of cooking over a fire without sounding too snakelike. |
| KR | cut | went for serrated cutting for maximal contrastiveness with ‘sharp’. |
| KR | deer | I’ve seen a lot of deer and never heard a deer sound other than cracking underbrush. Focused on representing their manner of motion but was very tempted to whistle some idyllic nature music„ maybe some Tchaikovsky flute piece. |
| KR | dull | tried for empty/hollow sound. To articulate this one the mouth assumes a very open shape- no articulators are tense (which might feel more pointed, or high spatial frequency), which I hope will make for a low spatial frequency (dull) embodied experience. |
| KR | eat | trying to be kind of generic and contrastive with ‘meat’ and ‘fruit’ |
| KR | fire | the white noise roar of a toasty fire. |
| KR | fruit | embarrassing/goofy though it may be, this utterance seemed to effectively convey biting into a juicy fruit in a manner that was not very mistakable for meat (I hope). |
| KR | gather | Iconically representing the repetitive nature of this temporally unfolding process. |
| KR | good | positive affect, /l/ consonant gets mouth close to smile shape. high F0. Hoping that wordiness of the utterance connotes a property rather than a verb or noun which are more easily iconicized in an utterance. |
| KR | hide | tried to convey startle in the abruptness of the utterance and a need to not be noticed with the voiceless/whisper. |
| KR | hunt | Wanting to be aggressive but needing to contrast with ‘pound’, aimed for more of a bow and arrow or dartlike sound- whiff?. |
| KR | knife | tried to take my serrated ‘cut’ sound and make it sound more nouny by giving it more syllabic structure (and vowels) and repeating the syllable. |
| KR | man | lower F0 with similar pseudosyllable structure to the other people words- hoping that judges might use this pseudosyntax to narrow down the options. |
| KR | many | multiple discrete syllables (very similar to ‘one’) individuated but repeated to represent a number of objects. |
| KR | meat | Tried to make this eating sound extra rich and hearty, and probably a little extra gross. |
| KR | one | a single discrete syllable with a stop to cleanly define it. High vowel for diminutive effect (to represent small magnitude). |
| KR | pound | an abrupt and loud utterance with a voiced stop symbolizing an impact and an engma for reverberation after the strike. |
| KR | rock | the sound of rocks knocking. |
| KR | sharp | relatively unobstructed consonants. |
| KR | sleep | snore without sounding too much like a tiger snarl. |
| KR | small | high F0 |
| KR | snake | instructions in the call were to not use ‘ssss’. I felt like the timing of this trill could represent either the flicking of a snake tongue or the rattle of a rattlesnake. |
| KR | that | Among others Woodworth 1991 and unpublished work by McCormick and Nygaard (2013 poster) spatial prepositions in 17 languages finds formant structure is systematically related to meaning, and duration and amplitude are related to native speakers judgments of meaning |
| KR | this | Among others Woodworth (1991) and McCormick and Nygaard (2013 poster) spatial prepositions in 17 languages finds formant structure is systematically related to meaning, and duration and amplitude are related to native speakers judgments of meaning |
| KR | tiger | snarl, or something like it. |
| KR | water | drip drip sound. |
| KR | woman | higher F0 than man with similar pseudosyllable structure to the other people words. |
| LJ | bad | The pitch rises at the end of this word to signal fear or alarm. The unrounded, low, back vowel is also one typically present in screams or sighs of displeasure. |
| LJ | big | I started with a very small lip opening and opened to a larger, more relaxed one. I chose to include an “h” because that sound also requires a wide open vocal tract. |
| LJ | child | As many people give cute pet-names to babies, so I tried to with the word “child.” The unvoiced consonants are gentle, and the repetition of the sound is reminiscent of something affectionate. |
| LJ | cook | Cooking combines a lot of different ingredients, and so this vocalization has a bunch of different components. The pitch varied up and down to also signify this diversity. |
| LJ | cut | “Cut” is a verb that requires a change in state, so there are two parts to the vocalization: “before” and “after” the cut. It is high pitched, quick, and abrupt. I tried to imitate an impact with the plosive “p.” |
| LJ | deer | Deer are herbivores and not dangerous to humans, so it makes sense that they would have an unthreatening vocalization. The “m” sound and rounded vowels make the changes between the sounds gradual. |
| LJ | dull | Oppositely from my vocalization for _sharp,” my interpretation of _dull” has no plosives. If a knife were sharp, it would be able to cut quickly, but as the knife here is dull, it is a longer vocalization and is not able to come straight to a _point” (aka a plosive). |
| LJ | eat | The first part of this vocalization is a smacking of the lips, which is followed by a vowel. The lips-smacking is reminiscent of eating, followed by a sound of contentment. |
| LJ | fire | The beginning sounds resemble a crackling fire, while the latter sound of the vocalization is a vowel. I imagined that vowel to be the sound one would make due to the pain of a burn. |
| LJ | fruit | My pitch is deep to seem similar to earthiness or grounded-ness. I chose articulators at the front of the mouth because those are commonly used in eating. |
| LJ | gather | The “sh” begins the vocalization with the lips outstretched forward. The vowel moves the focus back into the mouth, and then the “ng” at the end goes even further back into the person’s throat. In other words, the articulators go from farther to closer to the speaker as the word progresses, like gathering. |
| LJ | good | My pitch was higher and fell later in the word, imitating how I think of a coo directed at a baby or a cute animal. The final two vowel sounds are both front and unrounded, which feels a little like a smile. |
| LJ | hide | The most important things here are the unvoiced consonants. I tried to make this word as unobtrusive and quiet as possible. Suprasegmental features, especially distincetive quietness, seem important here. |
| LJ | hunt | Hunting is visceral, and so the vocalization is similar to a shout. The “k” is meant to make the word sound a little more violent. |
| LJ | knife | I tried to call upon the sharpest sounds here, specifically “z” and “k.” The addition of air make the word a little more like a spit, and as a result hopefully sounds more foreboding. |
| LJ | man | The sounds here, consonants and vowels, are very “sturdy.” My lower pitch is also meant to come off as masculine. |
| LJ | many | A vocalization with repetition seemed to be the best way to express “many, as opposed to one” in this case. I chose to make the repeated segment be two syllables instead of one, but that was mostly arbitrary, as was the picking of the sounds themselves. |
| LJ | meat | My pitch is very deep here to indicate that one of the main reasons to talk about meat is to eat it. This involves swallowing and also feelings deep in your belly. |
| LJ | one | I wanted the beginning and ending sound of this vocalization to be the same, to give it mainly one note. I do not vary in pitch or volume, reinforcing the idea of “oneness.” |
| LJ | pound | I chose something repetitive to mimic the repetitive nature of pounding. “B” seemed like a good plosive consonant that also seemed like something was hitting or being hit. |
| LJ | rock | This vocalization begins and ends with two plosive sounds to make it seem more “concrete.” It is spoken with a quite low pitch and seems to have some weight. |
| LJ | sharp | The “t” at the end of this vocalization is similar to a cut or a prick from something sharp. The ingressive airstream the beginning could mean a lot of things, but I created it to be like the intake of breath that one has when he or she is being cautious. |
| LJ | sleep | This vocalization is a bit breathy. The sounds are all relaxed, and there is nothing to impede the airflow at any time, hopefully mimicking sleep. |
| LJ | small | The vocalization begins with “m,” which is bilabial and minimizes space between the lips. The vowel opens the lips briefly but bring them back together quickly to a very small lip opening. I made sure the duration was quick to indicate “smallness” as well. |
| LJ | snake | The most distinctive characteristic of snakes is how they slither, so this vocalization tries to illustrate that. For this reason too, it is unvoiced. |
| LJ | that | My pitch rises and volume increases during this word to show that I am referring to something far from myself. |
| LJ | this | This is a single syllable because of its potential common usage. The lowering of pitch settles the word downwards and grounds it near myself. |
| LJ | tiger | During the development of protolanguages, animals like tigers probably defined by how dangerous they were to communities. My use of “k” here is the same as in my vocalization for “tiger”: it adds abruptness. |
| LJ | water | This vocalization kind of comes from gargling water in one’s mouth. It produces a consistent airstream, but it still has some friction. It sounds like water running over rocks in a stream. |
| LJ | woman | The “L” sound is meant to be not abrupt and flowing, like stereotypes of femininity. The pitch is relatively high and a little bit like singing or chanting. |
| MS | bad | In contrast, the pursed lips that come with a rounded vowel, and the closing off in a bilabial nasal coda are meant to be iconic of closedness or dismissiveness, which is how people typically respond to bad things. |
| MS | big | The low back vowel is meant to be iconic of largeness, as is length of the vowel, and the glide causes the mouth to keep opening, ‘bigger and bigger,’ as it were. |
| MS | child | Slightly higher pitch than women, iconic of the high pitch range of children, and also rising intonation at the end, representative of the questioning or submissive tone of young humans. |
| MS | cook | When asked about the meaning, both actors seemed to attach to the idea of a boiling pot, hence the bilabial onset (bubbles bursting). The labiodental fricative is from the hissing sounds involved in cooking with fire. This verb is a reduplication of the noun ‘fire.’ |
| MS | cut | The actors agreed on a short, high front vowel, and some kind of voiceless fricative. The initial fricative length may be iconic of the time it takes to make a cut (rather than a stab), and the velar coda of something sharp. This verb is a reduplication of ‘knife.’ |
| MS | deer | See ‘hide’ above. |
| MS | dull | The actors and we agreed that a dull implement was more likely to have a poundy (rather than cutty) sound, shown in the reverby nasel onset. |
| MS | eat | The actors agreed on back vowel, and one that involved a wide mouth. We like the muscular iconicity of starting with the tongue towards the front of the mouth with an alveolar liquid, and having the place of articulation move back to the back of the mouth, with velar fricative, simulating the act of swallowing food. This verb is a reduplication of the noun ‘meat.’ |
| MS | fire | When asked about the meaning, both actors seemed to attach to the idea of a boiling pot, hence the bilabial onset (bubbles bursting). The labiodental fricative is from the hissing sounds involved in cooking with fire. This verb is a reduplication of the noun ‘fire.’ |
| MS | fruit | This verb is a reduplication of the noun ‘fruit.’ We had no strong top down motivations for this verb or noun, but our actors insisted that some kind of /im/ needed to be in there, for the fruit, ‘because it gets all over your face.’ The wide-mouthed high front vowel may be iconic of a mouth open to eat. |
| MS | gather | This verb is a reduplication of the noun ‘fruit.’ We had no strong top down motivations for this verb or noun, but our actors insisted that some kind of /im/ needed to be in there, for the fruit, ‘because it gets all over your face.’ The wide-mouthed high front vowel may be iconic of a mouth open to eat. |
| MS | good | The wide mouthed vowel is meant to be iconic of smiling, and the rising intonation is meant to allude to openness. Pitch height may also fit in with the HIGH = GOOD metaphor, even though there’s nothing really high about a high vowel. |
| MS | hide | This verb is a partial reduplication of the noun ‘deer.’ The length of the onset, leading through a brief nucleus to an almost invisible, unreleased glottal stop coda, is meant to be iconic of the motion to get to a hiding place and then disappearing. |
| MS | hunt | This verb is a partial reduplication of the noun ‘tiger.’ For tiger, our actors like various trills, alveolar and uvular, so we chose as our coda the only trill we were able to produce. The velar onset comes from the sharpness of a tiger tooth or spear or arrow. |
| MS | knife | The actors agreed on a short, high front vowel, and some kind of voiceless fricative. The initial fricative length may be iconic of the time it takes to make a cut (rather than a stab), and the velar coda of something sharp. This verb is a reduplication of ‘knife.’ |
| MS | man | The actors insisted on a grunting, caveman sound for this noun, whence our low back vowel. The low pitch is meant to be iconic of men’s deeper voices. The switch from VCV to CVC is not motivated beyond wanting to have a different structure for human nouns. (Man, woman and child share similar phonology, differing only in vowel quality and intonation.) |
| MS | many | And then repeated it twice. Once might have sufficed, but single reduplication had already been used, and it might have been interpreted as dual, which some languages have a separate term for. |
| MS | meat | The actors agreed on back vowel, and one that involved a wide mouth. We like the muscular iconicity of starting with the tongue towards the front of the mouth with an alveolar liquid, and having the place of articulation move back to the back of the mouth, with velar fricative, simulating the act of swallowing food. This verb is a reduplication of the noun ‘meat.’ |
| MS | one | We just chose a short CV syllable for this. |
| MS | pound | The actors pounded mostly on their chest for this movement, and gave low back rounded vowels as a vocal feature that best describes their intuition. We thought the nasal vocalic onset and nasal coda was nicely iconic of the reverberations in pounding. This verb is a reduplication of ‘rock.’ |
| MS | rock | The actors pounded mostly on their chest for this movement, and gave low back rounded vowels as a vocal feature that best describes their intuition. We thought the nasal vocalic onset and nasal coda was nicely iconic of the reverberations in pounding. This verb is a reduplication of ‘rock.’ |
| MS | sharp | The velar onset is also meant to be iconic of sharpness. Honestly this one was hard. I guess they can’t all be winners. Maybe the unreleased glottal coda is iconic of how you would stop releasing your breath if you got murdered by a sharp thing? |
| MS | sleep | The actors alternated between various iconic interpretations of snoring, but we liked the glottal release, relaxed central vowel and effortless velar nasal qualities as iconic of the natural breathing state during sleep. This verb is not a reduplication of any noun, but follows the reduplication pattern of the other verbs. |
| MS | small | By contrast, small uses a short, high, front vowel, all on it’s lonesome, all meant to be iconic of littleness. |
| MS | snake | It was difficult to avoid the ‘No Words’ rule for this noun, regarding conventionalizations, as the conventionalized /s/ sound is also the clearest sound symbolic phoneme to represent its warning exhalation, or even its sliding, unbroken method of locomotion, which is the only other notable characteristic we could think of for snakes. So we went with voiceless glottal and labiodental fricatives. |
| MS | that | In contrast, greater distance fits with lower vowel and lower pitch. The vowel is also longer, mean to be iconic of greater distance. |
| MS | this | The thread of higher pitch/high front vowel/shorter length and smaller size fits here, too, with smaller size being smaller distance from the speaker. |
| MS | tiger | This verb is a partial reduplication of the noun ‘tiger.’ For tiger, our actors like various trills, alveolar and uvular, so we chose as our coda the only trill we were able to produce. The velar onset comes from the sharpness of a tiger tooth or spear or arrow. |
| MS | water | The actors jumped around between several instantiations of water: the dripping sound of rain, the gulping of drinking water and the flowing of river water. Still, they agreed on some kind of fricative, and the closing bilabial nasal is meant to be iconic of closing the mouth to swallow. |
| MS | woman | Besides grunting like stereotypical cavemen, though, our actors were remarkably unwilling to make any expressions of sex or gender differences, claiming that they were working hard on getting past all these stereotypes of gender normativity. This is Eugene, after all, so, liberals. Women do have higher pitches than men, and un-rounding the vowel seemed to naturally covary with raising the pitch. |
| NC | bad | we have chosen a humming sound, spontaneously produced at a lower pitch, following the conceptual metaphor of “bad is down”. |
| NC | big | as pointed by a group of scholars, big size is usually associated with low back vowels, voiced consonants, low tone (Ohala 1997), characterised with a lower pitch, longer duration, and higher intensity than its opposite “small” (Nygaard, Herold & Namy 2009). We have relied on these sounds and also tried to express some kind of awe. |
| NC | child | in this case we have decided to rely on a sound that would be similar to the sounds of motherese, as such an exaggerated intonation is commonly associated with children; we have also relied on sounds associated with diminutive meaning, i.e. high tones and repeated syllables (Perlman & Cain in press). |
| NC | cook | as “cooking” was connected with both “fire” and “eating”, we have decided to combine the sounds we have previously designed for “fire” and “eat”. |
| NC | cut | in “cut” we have relied on sounds similar to that associated previously with “sharp”; we have also decided to make it shorter than “knife” to make it seem more dynamic (as action is more dynamic than an object). |
| NC | deer | a moo-like sound. |
| NC | dull | round shapes – and thus the quality of being “dull” – can be associated with patterns of voiced sonorants combined with open, rounded vowels (Ahlner & Zlatev 2011 in: Perlman & Cain in press); we have relied on these sounds and also tried to make “dull” sound less alert than “sharp”. |
| NC | eat | in this case we have decided to rely on the imitation sound of eating (squelching, clicking the tongue). |
| NC | fire | as fire has been an important and a dangerous element of life, we have relied on a spontaneous sound that we associated with surprise and awe. |
| NC | fruit | to distinguish it from “meat”, we have relied on a pop-like sound (which for us evoked the associations with picking fruit) and combined it with the sound we have previously designed for “eat”. |
| NC | gather | gathering comes with repetition, so here we have relied on repeating the same sound at quite a dynamic rate. The sound itself [???] |
| NC | good | Unlike “pound”, however, “gather” sounds lighter, as it is not so much about force (and thus volume), but about skill (quickness, lightness). |
| NC | hide | to hide is to become invisible and inaudible, so we have relied on low voice volume, combined it with voiceless consonants, and tried to produce the sound in a whisper-like fashion. |
| NC | hunt | as hunting was an important event, requiring much attention and mobilisation from community members, we have decided to make the sound quite loud and energetic; our choice was a short, repeated whistle. |
| NC | knife | in this case we have relied on sounds similar to those used in “sharp”; however, we have decided to both link it as well as make is easily distinguishable with “cut” (as the two are quite connected); we have decided to make “knife” a bit longer than “cut”, to make it less dynamic (as object is less dynamic than an action). |
| NC | man | in this case we have relied on a humming sound made in a low man-like tone. |
| NC | many | we have chosen a random, short sound (a click) to correspond to “one”, and then used it repeatedly to designate “many”. This kind of correspondence (i.e. repeating the same sound to convey an idea of prolongation, repetition or higher number of sth) has been observed, for example, by Perlman and Cain in the case of vocal representations for “fast-slow” and “many-few” (in press). |
| NC | meat | in this case we have combined an aggressive, roar-like sound with the sound we have previously designed for “eat”. |
| NC | one | we have chosen a random, short sound (a click) to correspond to “one” and then used it repeatedly to designate “many”. This kind of correspondence (i.e. repeating the same sound to convey an idea of prolongation, repetition or higher number of sth) has been observed, for example, by Perlman and Cain in the case of vocal representations for “fast-slow” and “many-few” (in press). |
| NC | pound | in this case we have relied on imitation, relying on plosives and exaggerated mouth movement; we have also repeated the sound, as pounding often comes as a repeated action. |
| NC | rock | in this case we have relied on a sound imitating the sound of pressing one stone against another, as our main association with “rock” was stones used in tool- or fire-making. |
| NC | sharp | in the sharp-dull pair we have relied on the so-called “bouba-kiki”effect, which originally refers to phonemic representations of the qualities of shape (Perlman & Cain in press). Though “sharpness” and “dullness” are not shapes per se, the properties of being sharp or dull have much in common with surface structure and contour. Angular shapes – and thus the quality of being “sharp” – can be associated with patterns of voiceless obstruent consonants alternated with front, closed and unrounded vowels; we have relied on these sounds and also tried to express some kind of alertness, as sharp things have to be managed with caution. |
| NC | sleep | in this case we have decided to rely on the imitation sound of sleeping (or, actually, snoring). |
| NC | small | small size is usually associated with high front vowels, voiceless consonants and lower tone (Ohala 1997); we have relied on these sounds as well as tried to make it short (small) to designate “small”; we also aimed at contrasting it with its antonym from the set, i.e. “big”. |
| NC | snake | a hiss-like sound. |
| NC | that | Tanz (1971), who examined indexical words used to express proximity and distance (e.g. English “here” and “there”, found a strong tendency for languages to express close distance with front vowels and far distance with back vowels. Analogously to the pair above, we have chosen a short sound with a front vowel to correspond to “this” and recorded a similar sound with back vowel and prolonged for “that”, so as to mark the difference in length and distance between the two words. |
| NC | this | Tanz (1971), who examined indexical words used to express proximity and distance (e.g. English “here” and “there”, found a strong tendency for languages to express close distance with front vowels and far distance with back vowels. Analogously to the pair above, we have chosen a short sound with a front vowel to correspond to “this” and recorded a similar sound with back vowel and prolonged for “that”, so as to mark the difference in length and distance between the two words. |
| NC | tiger | a growl/roar-like sound. |
| NC | water | in this case we have relied on a sound that was to imitate the sound of flowing water, and tried to combine it with a liquid consonant. |
| NC | woman | in this case we have relied on a humming sound made in a higher, more woman-like tone, to distinguish it from “man”. |
| SU | bad | sound of disapproval |
| SU | big | sound of amazement |
| SU | child | sound of crying |
| SU | cook | bubbles popping like something boiling, then sound of enjoyment |
| SU | cut | meant to sound like a sharp object coming down on a target |
| SU | deer | meant to imitate prancing around |
| SU | dull | not necessarily something in particular but the opposite of something sharp |
| SU | eat | The sound was meant to imitate an exaggeration of a large bite of food |
| SU | fire | sound of using flint and steel to start a fire |
| SU | fruit | sound of squashing fruit |
| SU | gather | breathing in, collecting |
| SU | good | sound of approval |
| SU | hide | pulling cover over |
| SU | hunt | shot and kill |
| SU | knife | sound of something metallic |
| SU | man | grunt to represent deeper vocie |
| SU | many | sound of incredulity at a large amount |
| SU | meat | sound of eating |
| SU | one | a unity |
| SU | pound | hard attack, hard ejective |
| SU | rock | sound of somethign hard hitting something |
| SU | sharp | something metallic |
| SU | sleep | The sound was meant to imitate a snore, a common sound associated with sleeping |
| SU | small | small pitched sound |
| SU | snake | onomatopoeic sound of snake |
| SU | that | somewhat arbitrary |
| SU | this | somewhat arbitrary |
| SU | tiger | sound of a jungle cat as best made by my throat |
| SU | water | sound of water drops |
| SU | woman | sound of affection |
| TM | bad | the lower tone conveys seriousness/badness, and [f] sounds harsh and negative to us, so it is repeated twice |
| TM | big | the back vowel [u] sounds big, especially when lengthened |
| TM | child | the [ti] ending functions as a diminutive because voiceless stops and front vowels sound small |
| TM | cook | this was based on our utterance for “fire” since cooking involves fire; we just added an [h] to make “fire” into “cook” so that they are different. See tmnd_fire for the reasoning behind it |
| TM | cut | the velar sounds at the end, as well as the front vowels, convey a sharpness appropriate for cutting |
| TM | deer | the partial reduplication (of the small-sounding voiceless stops and front vowels) is meant to convey a deer lightly running through the woods |
| TM | dull | the single, short syllable with a central vowel is meant to sound dull |
| TM | eat | the implosive is meant to suggest ingesting matter because it sounds like a gulp |
| TM | fire | the fricatives mimic the hissing sound of fire |
| TM | fruit | the [ul] and [aba] components are meant to emulate the feeling of sweet fruit in the mouth |
| TM | gather | the non-exact reduplication conveys how gathering is repetitive but doesn’t repeat the same motion exactly since you gather different items from different places. |
| TM | good | the higher tone conveys cheerfulness and goodness |
| TM | hide | the ingressive conveys nervousness and a desire to escape |
| TM | hunt | meant to convey the sound of a spear being thrown [hi], flying through the air [fwu], and hitting a target [put]. Together they make [hifwuput] |
| TM | knife | the velar sounds, as well as the front vowels, convey a sharpness appropriate for a knife |
| TM | man | the low tone mimics a man’s voice, and the word is short and simple because it is such a basic word |
| TM | many | formed by reduplication of “one” since “many” = multiple “one”s |
| TM | meat | the nasals mimic the sound of chewing |
| TM | one | a short, simple word to convey singleness and the basicness of the concept |
| TM | pound | the reduplication conveys the repetitiveness, and the sounds chosen mimic the noise of pounding two rocks together |
| TM | rock | formed from half of “pound” since pounding involves rocks, and the voiced stops and back vowels sound big and dull, like a rock |
| TM | sharp | the velar sounds, as well as the front vowels and voiceless alveolar stop, convey sharpness |
| TM | sleep | the fricatives and falling tones mimic the sound of a sleeping person’s breathing |
| TM | small | the voiceless stops and front vowels sound small |
| TM | snake | the initial [s] mimics hissing, then the [l]’s convey slipperiness, and the final [h] sounds sinister |
| TM | that | “that” is similar to “this” but with a falling tone and a diphthong of two back vowels to convey _over there”-ness |
| TM | this | “this” is similar to “that” but with a rising tone and a diphthong that contains a front vowel to convey nearness |
| TM | tiger | the fricatives sound sinister, particularly the geminated, growl-like [h] at the end |
| TM | water | the post-alveolar fricatives mimic the sound of water gently falling |
| TM | woman | Like “man” but with a front vowel at the start, to convey that women are smaller, and with a high tone to mimic a woman’s voice |
